# From design to validation of CRISPR/gRNA primers towards genome editing

**DOI:** 10.6026/97320630018471

**Published:** 2022-05-31

**Authors:** Mohd Rizwan Jamee, Zubaida Ansari, Mohammad Irfan Qureshi

**Affiliations:** 1 Department of Biotechnology, Jamia Millia Islamia, New Delhi – 110025, India; 2 Centre for Interdisciplinary Research in Basic Science, Jamia Milia Islamia, New Delhi – 110025, India; 3 International Centre for Genetic Engineering and Biotechnology, New Delhi ˗ 110067, India

**Keywords:** CRISPR, CRISPdirect, primers, knock out, soluble starch synthase (SSS)

## Abstract

CRISPR (Clustered Regularly Interspaced Short Palindromic Repeats)/Cas9 (CRISPR-associated system) is used to edit specific genomic sequences with precision and efficacy. There are many online platforms/software for the design of gRNAs and related primers. However, there are concerns in design regarding off-site deletions besides knocking out sequences in the target genes. Nonetheless, a well known robust platform for CRISPR/gRNA primers design is CRISPRdirect. We demonstrate the use of this tool in the design of CRISPR/gRNA primers for soluble starch synthases (SSS) II-1, 2, and 3 genes in the Oryza sativa genome followed by the PCR-mediated amplification of SSS genes with corresponding confirmation towards genome editing having improved phenotype features.

## Background:

Clustered Regularly Interspaced Short Palindromic Repeats (CRISPR)/Cas9 endonuclease is a genomic editing tool. CRISPR/Cas9 is known for its simplicity, specificity, and versatility [1-5]. The functions of endonuclease Cas9 could be utilized to delete a specific site(s) of a gene using an engineered sequence single guide RNA (gRNA). The gRNA usually has 20 nucleotides against a specific target sequence in the genome [5]. Mutations in soluble starch synthases (SSS) II [6-9] have altered the starch biosynthesis pathway in one way or the other in rice. Variation in SSS isoform profiles could affect the starch biosynthesis pathway and control phenotypes in the natural varieties of rice [10]. Thus, design of specific CRISPR/gRNA primers is a challenge.

There are several online platforms/software offering services to design gRNAs and related primers (Table 1 - see PDF). We used the CRISPRdirect (http://crispr.dbcls.jp/) [11] platform to select target sites of gRNA and NGG as PAM sites in SSS II (1, 2, and 3) genes with possible off-target sites. The CRISPRdirect examined the whole genome template of Oryza sativa at length width of 20-mer, 12-mer and 8-mer adjacent to the PAM (protospacer adjacent motif) of the flanking region [11]. Therefore, it is of interest to describe the design of CRISPR/gRNA primers [11] for soluble starch synthases (SSS) II-1, 2, and 3 genes in the Oryza sativa genome using CRISPRdirect followed by the PCR-mediated amplification of SSS genes with corresponding confirmation.

## Material and Methods:

### Workflow:

The workflow in the CRISPR/gRNA primer design for engineering target sites in genomes using the CRISPRdirect software is shown in Figure 1(see PDF).

### Sequence search using BLAST:

Various DNA and protein sequence databases (NCBI, PDB, TAIR, Swissport, Rice Genome Annotation Project at MSU, etc.) are used for sequence search using BLASTN and BLASTX programs [33]. The rate likenesses among DNA and protein sequences were acquired utilizing MacVector programming (Acceleris, GmbH, Germany) and Bioedit programming (variant 7.25).

### Alignments of DNA sequences:

Alignments of the DNA sequences were completed using the Bioedit software (version 7.25) and ClustalW (version 2.0) program [34] at EMBL.

### Primer design:

The primers were designed using Mac-Vector (Acceleris, GmbH), Snap Gene (form 1.1.3), or using electronic programming, for example, Primer blast (http://www.ncbi.nlm.nih.gov/ instruments/ groundwork impact/list) and Primer 3 plus (https://primer3plus.com/) with default values. Oligo-analyzer programming (http://www.idtdna.com) was used to check for the probability of self or hetero-dimer development in the planned set of Primers. These Primers were confirmed for their uniqueness using BLASTN at the NCBI database. The primers were further combined using IDT, Germany, or Sigma-Aldrich, India.

### gRNA synthesis:

The target site to be knocked out was picked in the exonic region involved in gene expression. Thus, to pick the target sites in the cDNA sequence of the gene of interest was used. Both the forward and turn around single-stranded oligos of 20 ntds synthesized at IDT (USA) were used.

### Restriction map analysis:

Restriction maps of DNA fragments were generated using the Snap Gene software (version 1.1.3) to recognize the restriction sites in the required DNA sequence for either cloning into a range of vectors or for the construction of primers containing restriction sites.

### Polymerase Chain Reaction:

The amplification of target DNA sequence was performed using standard protocol with all required components (PCR mixture) including Taq polymerase, and pair of primers at overhang regions at the gRNA target sites.

## Results:

### Sequence search using BLAST:

A BLAST data for the target gene Soluble Starch Synthase (SSS) from Oryza sativa indica showed 8 closes matches with 100% query coverage, and 99.90% to 99.79% identity (Table 2 - see PDF). Distance tree for the data is shown in Figure 2(see PDF).

### Primers of CRISPR/Cas9 gRNA:

CRISPR primers and gRNA were designed for SSS II 1, 2 and 3 (Tables 3, 4, and 5 - see PDF) using CRISPRdirect (Figure 3 - see PDF). Primers for other required genes such as hygromycin resistant gene (hygromycin phos-phor-transferase, hpt) and Cas9 were also designed (Table 6). Next-generation sequences (NGS) were used for generating targeted sequences. Blast search likeness of SSS II-1, 2 and 3 (Table 3 - see PDF) gene sequences were completed at several DNA databases (PDB, NCBI, TAIR, Swissport, Rice Genome Explanation at MSU). Alignment of the SSS II sequences were completed using the bio edit software (version 7.25) and ClustalW (version 2.0) program [34] of EMBL. The majority of the primers of CRISPR gRNA for target specific sequences of SSS II were done either using the Mac-Vector (Acceleris, GmbH), CRISPRdirect, Snap Gene (form 1.1.3), or electronic programming, for example, Primer blast (http://www.ncbi.nlm.nih.gov/instruments/groundwork impact/list) and Primer 3 plus (https://primer3plus.com/) with default limitations. Oligo-analyzer programming (http://www.idtdna.com) was used to check for the probability of self or heterodimer development in the designed set of Primers of CRISPR Cas9. These Primers were confirmed for their uniqueness using BLASTN at the NCBI database.

## Discussion:

The CRISPR/Cas9 application is used for gene editing. Gene editing in the intronic region will have no impact. Targeting exon regions is essential for effective approach to delete functional segment of gene in CRISPR/Cas9-mediated genome editing. The gRNA can be changed by digesting the scaffold vector with an appropriate restriction enzyme to clone sgRNA. cDNA sequences of three OsSSSII-1, OsSSSII-2, and OsSSSII-3 genes were used in the CRISPRdirect program predict potential gRNAs. The CRISPRdirect identified few target sites which were promptly adjoining PAM sequences while checking the sense and antisense strands of both the cDNAs. The target sequence resembled 5'-N (20)- NGG-3' or 5'-CCN-N (20)- 3'. The target sequence which is 20 ntds, was available only near the PAM sequence which is 5'NGG3'. Both the forward and turn around single-stranded oligos of 20 ntds were obtained from IDT (USA). These data shows that CRISPRdirect [11] CRISPR-p [35] Cas-OF finder [16] and Cas-OT [36] are important tools for designing gRNAs and for predicting off targets. Design of primers of CRISPR/Cas9 gRNA using CRISPRdirect is simple and effective. The program generates a list of 20-22 bp spacers for targeted sequence for a gene with the search of PAMs (5'-NGG-3') on both gene strands. Thus, we describe the enhanced amylose rice (data not shown) using CRISPR/Cas9 tool targeting isoforms 1, 2 and 3 of SSSII. Hence, designed gRNA respective targeted SSSII-1, SSSII-2, and SSSII-3 in the Indica rice with transgenic-free homozygous SSSII mutant were generated. Therefore, food product made of flour with higher amylose content with resistant starch (RS) is possible from effective design of primers.

## Conclusion:

We describe the use of the CRISPRdirect tool in the design of CRISPR/gRNA primers for soluble starch synthases (SSS) II-1, 2, and 3 genes in the Oryza sativa genome followed by the PCR-mediated amplification of SSS with corresponding confirmation towards genome editing. Thus, the use of the CRISPRdirect tool in the design of CRISPR/gRNA primers towards genome editing having improved phenotype features is illustrated.
